# Improvement of Lung NET Management through Standardized Care—A Swiss Nationwide Observational Study

**DOI:** 10.3390/cancers15082270

**Published:** 2023-04-13

**Authors:** Moira Schmidlin, Samira M. Sadowski, Alexander Siebenhüner, Damian Wild, Emanuel Christ, Julie Refardt

**Affiliations:** 1 ENETS Center of Excellence for Neuroendocrine and Endocrine Tumors, University Hospital Basel, 4031 Basel, Switzerland; 2 Department of Clinical Research, University of Basel, 4031 Basel, Switzerland; 3 Endocrine Surgery, National Cancer Institute, Bethesda, MD 20892, USA; 4 Hirslanden Zurich AG, Clinic for Hematology and Oncology, 8032 Zurich, Switzerland; 5 Clinic for Medical Oncology and Hematology, University Hospital Zurich and University of Zurich, 8091 Zurich, Switzerland; 6 Division of Nuclear Medicine, University Hospital Basel, 4031 Basel, Switzerland

**Keywords:** typical carcinoid, atypical carcinoid, pulmonary carcinoid, registry, treatment, survival analysis

## Abstract

**Simple Summary:**

Patients with low- and intermediate-grade neuroendocrine tumors (NETs) of the lung, also called typical (TC) and atypical carcinoids (AC), are often managed in a very heterogenous way. This lack of standardized procedures, probably due to the rare occurrence of these tumors, was and is unfavorable for the affected patients. In 2015, the European NET society (ENETS) published new guidelines for best practice for TC and AC. Using data from the SwissNET registry, we wanted to check whether there was an impact of these guidelines on the diagnosis and treatment of the affected patients. As we could see, there was an improvement after 2016, in the sense of an increase in functional imaging and the determination of histopathological markers. Additionally, more systemic lymph node resections were conducted. These findings are encouraging; nevertheless there is room for further improvement in the management of these patients.

**Abstract:**

Typical (TC) and atypical carcinoids (AC) are the most common neuroendocrine tumors (NETs) of the lung. Because these tumors are rare, their management varies widely among Swiss centers. Our aim was to compare the management of Swiss patients before and after the publication of the expert consensus of the European Neuroendocrine Tumor Society (ENETS) in 2015. We used data from the Swiss NET registry from 2009 to 2021 with patients with TC and AC. Survival analysis was performed using the Kaplan–Meier method and log-rank test. Overall, 238 patients were included, 76% (180) thereof with TC and 24% (58) with AC, including 155 patients before and 83 patients after 2016. An increase in the use of functional imaging was observed, 16% (25) before and 35% (29) after 2016, *p* < 0.001. The presence of SST_2A_-receptors was determined more often: 32% (49 times) before 2016 and 47% (39 times) after, *p* = 0.019. Concerning therapy, higher removal of lymph nodes after 2016 was observed, 54% (83) before versus 78% (65) after, *p* < 0.001. Median overall survival for patients with AC was significantly shorter, with 89 months compared to 157 months for patients with TC, *p* < 0.001. While the implementation of a more standardized approach was observed over the years, there is still room for amelioration in the management of TC and AC in Switzerland.

## 1. Introduction

Neuroendocrine neoplasms (NENs) are rare tumors derived from neuroendocrine cells, which occur at several sites throughout the body. NENs are characterized by their usually homogenous overexpression of hormone receptors on their cell surface. A minority of NENs (10–30%) [1,2] have the ability to secrete biologically active peptides or amines and provoke clinical symptoms, such as diarrhea and flush, so-called functioning NENs. 

After the gastrointestinal tract, the lung is the second most affected organ, representing 30% of all NENs [3]. A common way of grouping lung NENs is to differentiate them into low-, intermediate-, and high-grade tumors, the first two commonly denotated as typical (TC) and atypical carcinoids (AC). TC and AC, summarized as pulmonary carcinoids (PC), tend to occur in the 40–60th year of life and are rather slow-growing tumors. AC are a more aggressive entity, with a higher likelihood of metastasizing to lymph nodes and distant organs, and have a reduced overall survival rate [4]. TC have a higher prevalence than AC, representing 80–90% of all PC [2,5]. 

The classification of lung NENs is based on the WHO criteria of 2004, including mitotic count and presence of necrosis. The use of the Ki67 proliferation rate is not commonly accepted but has been shown to be a useful addition to the established diagnostic criteria by Rindi et al. [6]. Although NENs are a rare tumor entity, an increasing incidence up to 6.98/100,000 has been observed in the past decades, occurring at all sites, stages and grades [7].

A recent nationwide study further revealed a heterogeneous approach with incomplete imaging and much missing data in pathological and labor-analytical workup [8]. Additionally, Singh et al. [9] showed a diagnostic delay for NEN patients as well as several unmet needs concerning the management of NEN. Probably the lack of routine in the management of PC and the limited number of randomized studies have led to this paucity in standardized procedures. Nevertheless, a prompt and precise diagnosis should lead to an appropriate therapy and thus an impact on prognosis. In 2015, the ENETS has published an expert consensus and recommendations for best practice concerning PC, as an attempt to improve the handling of such patients and therefore their outcome [2]. During the diagnostic proceeding, pathology is the most important asset in forming a PC diagnosis. The gold standard for imaging remains the contrast CT, but the often somatostatin-receptor (SST)-positive tumors and eventual metastasis can be easily detected with functional imaging, such as ^68^Ga-DOTATATE PET/CT or ^68^Ga-DOTATOC PET/CT. This aspect of SST-receptor-positivity is also used therapeutically by using peptide receptor radionuclide therapy (PRRT) or somatostatin analogues (SSA). Furthermore, a standardized approach for surgery, including systemic nodal dissection, is strongly advised. 

In this study, we aim at investigating whether diagnosis and therapy for PC in Switzerland has improved according to the recommendations of the ENETS 2015 guidelines.

## 2. Materials and Methods

### 2.1. Participants

The Swiss registry for NENs (SwissNET) was established in 2008 and prospectively includes data of patients with NEN at all sites and stages in Switzerland. All patients in the registry have provided their informed consent, and all cantonal Swiss ethics committees have approved the registry and its proceedings and regulations.

For this retrospective study, all adult patients of the Swiss NET registry with TC or AC of the lung from the beginning of 2009 until end of august 2021 were included. Patients had to have had at least one follow-up or a reported outcome after the first treatment. Patients without sufficient information regarding the treatment approach were excluded. Patients with tumorlets (size < 5 mm), mixed adenoma, well-differentiated neuroendocrine tumors (MANET), and/or diffuse idiopathic pulmonary cell hyperplasia (DIPNECH) were not included in the analysis. 

### 2.2. Staging

Tumor grade and stage were determined at time of diagnosis, using all available information, e.g., histopathological examination and imaging techniques. Classification and grading was conducted following the WHO criteria of 2004 (as proposed by the ENETS guidelines) and the seventh edition of the Union Internationale Contre le Cancer/American Joint Committee on Cancer (UICC/AJCC) TNM classification [2,10]. According to these guidelines, TC present fewer than 2 mitoses/2 mm^2^ and no necrosis, while AC show 2–10 mitoses/2 mm^2^ and/or points of necrosis. The Ki67 proliferation index is not a dependable source for separating TC from AC but can be used to separate PC from neuroendocrine carcinoma (NEC), especially in small or crushed biopsies. 

### 2.3. Statistics

To assess demographic and clinical data, we used descriptive statistics. Unless otherwise stated, all data are expressed as mean with standard deviation (SD) or median with interquartile range (IQR). The Pearson chi-squared test or Fisher’s exact test was used to determine potential association between categorical variables, and the Mann–Whitney U test was used for the potential association of continuous variables. Overall survival (OS) and progression-free survival (PFS) were calculated with the Kaplan–Meier method, using the log-rank test as a significance test. Univariate and multivariate Cox proportional hazard models were used to investigate independent predictors of OS and PFS. *p* values of <0.05 were chosen to indicate statistically significant differences. Missing data were not imputed, and the number of missing values was indicated. To evaluate practice change, patients diagnosed before and after 2016 were compared. All statistical analyses were conducted using SPSS version 28.0.1.0. Prism Graph Pad version 7 was used for graphs. 

## 3. Results

### 3.1. Patient Characteristics 

A total of 238 patients fulfilled the inclusion criteria, with the majority being treated in a tertiary-care hospital (for details, see Table 1). Sixty two percent (*n* = 147) of the included patients were female, with median (IQR) age 62.6 years (53.8–71.1). Forty percent (*n* = 95) of the PC were reported to have been diagnosed incidentally. Twenty-five patients (11%) had a functioning tumor, of which 19 patients suffered from carcinoid syndrome (76%) and five patients (20%) from Cushing syndrome. For one patient, the type of hormonal syndrome was not specified.

Seventy-six percent (*n* = 180 patients) of the PC were diagnosed as TC, while twenty-four percent (58 patients) were diagnosed as AC. Concerning patient characteristics, no difference was seen between the two tumor entities.

### 3.2. Diagnostic Evaluations

The main conventional imaging method was a CT scan (204 patients, 86%), followed by chest X-ray (111 patients, 47%) and MRI (31 patients, 13%). Concerning functional imaging, 33% underwent somatostatin-receptor-based imaging (79 patients), and 38% (91 patients) had an FDG PET/CT (for details see Table 2). Fourteen patients (5.8%) had both an FDG PET/CT and a ^68^Ga-DOTATATE PET/CT or ^68^Ga-DOTATOC PET/CT. 

Median (IQR) tumor size was 17 mm (12–29), with no difference in median tumor size between TC and AC (TC = 17 mm (range 12–25 mm), AC = 26.5 mm (range 13–45 mm), *p* = 0.109). At the time point of diagnosis, 53 patients had lymph node metastasis (N1 = 34 (14%), N2 = 17 (7%), N3 = 2 (1%)), and 23 patients (10%) had distant metastasis. Positive lymph nodes were more frequent at diagnosis in AC (26 patients, 45%) than in TC (27 patients, 15%), *p* < 0.001; the same was found for distant metastasis (AC = 14 (24%), TC = 9 (5%), *p* < 0.001). 

Concerning histopathological evaluation, Chromogranin-A staining was positive in 185 patients (78%) and Synaptophysin in 197 patients (83%). SST staining was only performed in 88 patients (37%), with SST subtype 2A (SST_2_)-receptor positivity in 45 patients (19%). The Ki67 proliferation index was measured in 156 patients (66%), with a median (IQR) of 2% (1–5) in TC and of 5% (5–13.5) in AC. 

Most of the patients had a stage Ia disease at time of diagnosis (118 patients, 50%). Twenty-four patients (10%) were already at stage IV at this point. Patients with TC had a statistically lower stage at the time point of diagnosis compared with patients with AC (*p* < 0.001). 

Of all patients, 21% (51) developed distant metastases with the main locations involving the liver (30 patients, 13%), bone (26 patients, 11%), and lung (12 patients, 5%). Biomarkers were assessed sparsely, with available values for Chromogranin A in only 28 patients (12%), for NSE in only 4 patients (2%), and 5-HIAA in plasma and urine in 9 patients (4%).

### 3.3. Therapy

For most PC patients (86%, 205 patients), surgery was the first therapeutical approach. In 82% (195 patients), the surgical intervention led to an R0 resection. Additionally, in 62% (148 patients), a lymph node resection was performed. The surgical procedures, at any point in the therapeutic approach, were classified as lobectomy in 40% (96 patients), wedge resection in 17% (40 patients), segmentectomy in 14% (33 patients), and bilobectomy in 6% (14 patients). In eight patients (3%), the procedure was simply classified as lung surgery. Furthermore, three bronchial resections were conducted (1%), two pneumectomies, two lingula resections and one sleeve resection. When comparing lobar versus sublobar resections, a difference between TC and AC could be stated, with more lobar resections in AC patients (*p* = 0.012).

Five percent (12 patients) had additional external radiation, seven percent (17 patients) received PRRT, and seven percent (17 patients) chemotherapy (for details see Table 3). In 17 patients (7%), SSA were given, and 12 patients (5%) received Everolimus, one of them in combination with Sunitinib. A few patients had an ablative therapy, such as radiofrequency ablation (RFA) (4 patients), embolization (2 patients), laser interstitial thermal therapy (LITT) (1 patient), and cryotherapy (1 patient). 

We could see that there was a significant difference in the first applied treatment comparing TC and AC. While in TC, surgery was the first treatment of choice in 161 patients (89%), in AC it was only in 44 patients (76%) (*p* < 0.001). In AC, additional therapies such as irradiation (TC = 3.2%; AC = 12.21%; *p* < 0.001), the use of PRRT (TC = 4.2%; AC = 13.22%; *p* < 0.001) or SSA (TC = 8.4%; AC = 9.16%; *p* = 0.012), chemotherapy (TC= 4.2%; AC= 13.22%; *p* < 0.001), targeted therapy (TC = 4.2%; AC = 8.14%, *p* = 0.002), and ablative therapy (TC = 3.2%; AC = 5.9%; *p* = 0.042) were used significantly more often. 

### 3.4. Outcome

Median (IQR) follow-up time was 49.7 months (16.5–84.8). At the end of follow-up, 66% (158 patients) were in complete remission or were discharged from follow-up. One patient was in partial remission, and thirteen had stable disease (5%). Ten patients had progressive disease (4%), and thirty-three (14%) died. Eight patients (3%) were lost to follow-up, and for fifteen patients, status at last follow-up was unknown. Overall, 28% of all patients with AC (16 patients) and 9% of all patients with TC (17 patients) died, marking a clear difference in mortality in the two tumor entities (*p* < 0.001). 

**Figure 1:**
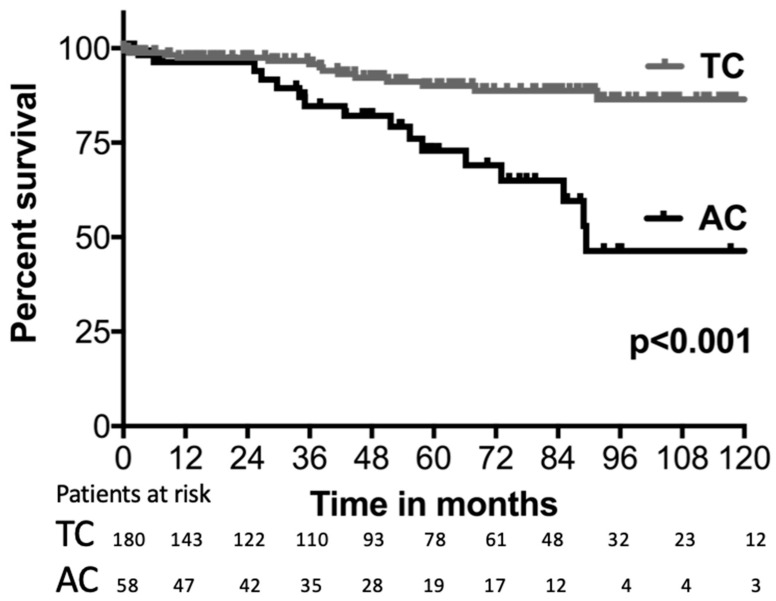
Overall survival. Kaplan–Meier survival curve comparing overall survival of patients with typical carcinoids (TC) and atypical carcinoids (AC). Only the first 10 years of follow-up are displayed.

**Figure 2:**
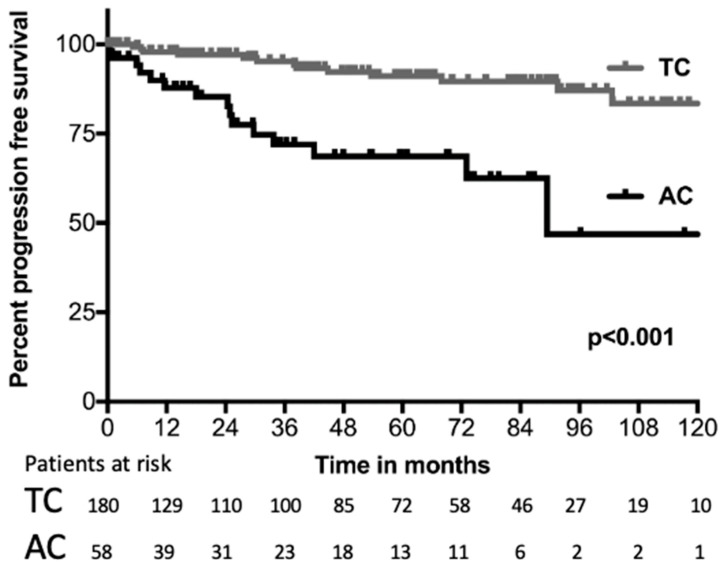
Progression-free survival. Kaplan–Meier distribution of progression-free survival, comparing patients with typical carcinoids (TC) and atypical carcinoids (AC). Only the first 10 years of PFS are displayed.

Comparing TC and AC, the median (95% CI) OS was significantly longer in TC patients, with 156.8 months (107.1–206.5) compared to 89.4 months (no CI) in AC patients, *p* < 0.001 (see Figure 1). Moreover, PFS was shorter for patients with AC, with 74 months (no CI), while the median was not reached for TC; *p* < 0.001 (see Figure 2). 

Univariate and multivariate analyses revealed no clinical variable or factor influencing the OS and PFS differences between TC and AC. The variables tested for were the Ki67 index, surgery type, surgical versus non-surgical therapeutical approach, sublobar versus lobar resection, the performance of a lymphadenectomy, and whether the treatment was conducted in a university hospital or not. 

### 3.5. Comparison before and after 2016

To check whether there was an improvement in diagnostic workup, management, and care of patients with PC after the publication of the ENETS guidelines in 2015, we divided our study population into two groups: one before 2016, containing 155 patients, and one after, containing 83 patients (reference date 31 March 2016) (see Table 4). The distribution of TC (before = 117; after = 63) and AC (before = 38; after = 20) in the two groups was similar (*p* = 0.94). 

In the diagnostic approach, an increase in the use of functional imaging was noted, such as ^68^Ga-DOTATATE PET/CT or ^68^Ga-DOTATOC PET/CT (before, 25 times (16%); after, 29 times (35%), *p* < 0.001). At the same time, the use of somatostatin receptor scintigraphy (SRS) (before, 13%; after, 1%, *p* < 0.001) and FDG PET/CT (before, 46%; after, 23%, *p* < 0.001) decreased. 

In addition, the potential presence of SST_2A_ receptors was determined more often after 2016 (49 times (32%) before, versus 39 times (47%) after, *p* = 0.019), as well as the Ki67 proliferation index (89 times (57%) before, versus 67 times (80%) after, *p* < 0.001).

Concerning therapy, there was only a change in the resection of lymph nodes, which was conducted more often after 2016. Before, 83 patients (54%) had a resection, and after, 65 (78%) did, *p* < 0.001. There was no difference in the first chosen treatment modality, in the type of surgery, or the use of additional treatment modalities. OS and PFS were approximately the same for the two groups (OS: *p* = 0.36, PFS: *p*= 0.26). 

## 4. Discussion

Our study has several main findings: first, we were able to confirm the more malignant behavior of AC compared to TC, which leads to a higher number of affected lymph nodes, more remote metastases, and a higher stage at time point of diagnosis. Accordingly, OS and PFS were decreased for AC compared to TC patients. Secondly, we were able to show that guidelines do have an effect on the evaluation of PC, leading to higher rates of ^68^Ga-DOTATATE PET/CT or ^68^Ga-DOTATOC PET/CT imaging, as well as SST_2A_ receptor and Ki67 proliferation index determination. Treatment was also influenced, with a higher rate of additional nodal dissection in TC and AC. 

Even though PC were considered benign lesions for a long time, sufficient evidence is available currently that shows their potential to metastasize and spread locally, especially in AC [4,11,12], which we were able to confirm with our data. As a result, many studies point to significantly reduced survival in AC, as did, for example, Forde et al. in metastasized or advanced PC [13]. Moreover, in PC of a lower stage, decreased OS and PFS in AC compared to TC was also observed [14]. The fact that TC and AC can be separated in such a distinctive way, histologically and concerning their prognosis, shows the validity of the use of WHO classification.

In our cohort, we could see a prevalence of 24% of AC, which is more than the generally described 10–20%. While other studies show a similar distribution of the two tumor entities [15,16], an overestimation of AC due to missing pathological information in our cohort could have taken place. 

The gold standard for radiological imaging at diagnosis is a contrast CT [2], whereas functional imaging also has a high sensitivity for showing primary tumors and metastases. ^68^Ga-DOTATATE PET/CT or ^68^Ga-DOTATOC PET/CT are more sensitive and thus preferable to SRS. In addition, these procedures, using the potential presence of SSTRs, help to predict the effect of possible therapy with SSA or PRRT (theranostic approach). While the increased use of ^68^Ga-DOTATATE PET/CT or ^68^Ga-DOTATOC PET/CT and decreased use of SRS is certainly also partly explained by technological improvement and higher availability, this cannot be said for FDG PET/CT. Since FDG PET/CT is used especially for poorly differentiated forms of PC and NEC, the growing understanding and earlier recognition of the low and intermediate PC likely led to its lesser use after 2016 in our cohort. 

Pathology is the gold standard for the assessment of diagnosis for PC [2], including mitotic count, necrosis, and the Ki67 proliferation index. Although other studies reported the Ki67 index to be a prognostic factor [6,17], this was not true in our cohort. While we saw an increase in the assessment of the Ki67 labeling index after 2016, the case number might have been too low to show such an association, since in 34% of the cohort, no Ki67 value was measured. In addition, almost no indication of the presence of necrosis was obtained, which indicates the need for further emphasis on the importance of standardized pathology reports [18]. At the same time, the identification of SST_2A_ receptors using immunohistology did increase but was still missing in 53% of the samples after 2016.

In the current guidelines [2], the focus for deciding for the surgical approach is the anatomical localization of the tumor—an information that was unfortunately not available in our database. In localized disease, the recommended therapy is surgery, if possible, in the form of a complete anatomic resection if the tumor is peripheral. This includes a nodal dissection. In general, lung-sparing procedures should be favored over pneumonectomy. However, there is a disagreement on the impact of sublobar versus lobar resection on outcome. Our analysis showed no influence on OS or PFS between these two interventions. Recent studies [19,20,21,22], including a SEER database analysis [11], also showed no influence on prognosis or OS. Other studies, however, conclude that non-anatomic resections were related to a higher risk of developing distant metastases [4] or a higher recurrence rate [23]. It follows that more prospective and randomized studies are necessary to evaluate the needed extent of appropriate surgical resection. Interestingly, more lobar resections in AC patients were observed after 2016 in our study. An explanation for this could be the rising awareness of the more malignant nature of AC.

Many studies show a significant influence of lymph node affection on survival [24,25]; for example, Cardillo et al. [24] stated that prognosis in PC was even more related to nodal status than histologic subtype. Although there are overall limited data on the influence on OS, lymphadenectomy is recommended by most authors [24,25,26] as well as in the ENETS guidelines [2], where a systemic nodal dissection is advised in TC and AC. Our findings coincide with those of Brown et al. [19], indicating that lymphadenectomy is not an independent predictor of OS or PFS. They, too, state that it should be nevertheless performed, because of the potential prognostic influence of lymph node upstaging. Accordingly, an increase in lymph node resection after 2016 was observed in our cohort. 

As with every study working with a registry, we had limiting factors such as missing and inconclusive data and non-available important variables. An example of this would be the missing indication of tumor localization, which is important for therapy evaluation and prognosis [2,27]. Every database is reliant on reporting, which can only be controlled to a certain extent. In addition, the data are observational and therefore at risk for selection bias. Additionally, the heterogenous diagnostic and therapeutic approach for the patients led to various different submissions and a rather small sample size in the subgroups. We were also not able to create detailed timelines in the initial diagnostic and therapeutic procedures. Moreover, although the ENETS guidelines are well known and widely adopted in Switzerland, they are not mandatory for participating centers, which makes their impact evaluation challenging. Nevertheless, through our careful review and patient selection, our study gives an important insight into the management of this rare tumor entity.

## 5. Conclusions

In summary, our retrospective analysis showed an amelioration of diagnostic management and treatment of TC and AC through a more standardized approach. Although no influence on the OS or PFS was seen, implementing existing guidelines will further improve the management of these rare tumors.

## Figures and Tables

**Table 1:** Baseline characteristics.

	All (*n* = 238)	Typical Carcinoid (*n* = 180)	Atypical Carcinoid (*n* = 58)	*p* Value	
**Age at diagnosis (years)**	62.6 (53.8–71.1)	62.3 (52.6–70.8)	64.1 (55.3–73.6)	0.65	
**Sex (female)**	147 (61.8%)	112 (62.2%)	35 (60.3%)	0.79	
**Functioning tumor**	25 (10.5%)	19 (10.5%)	6 (10.3%)	0.73	
**Tumor incidentally found**	95 (39.9%)	73 (40.5%)	22 (37.9%)	0.31	
**Treatment at University Hospital**	134 (56.3%)	108 (60%)	26 (44.8%)	**0.043**	
**Size of primary lesion at diagnosis (mm)**	17 (12–29)	17 (12–25)	26.5 (13–45)	0.10	(a)
**Ki67**	3 (1.6–5)	2 (1–5)	5 (5–13.5)	**<0.001**	(b)
**Positive lymph nodes at diagnosis**				**<0.001**	
N0	180 (75.6%)	150 (83.3%)	30 (51.7%)		
N1	34 (14.3%)	16 (8.8%)	18 (31%)		
N2	17 (7.1%)	10 (5.5%)	7 (12%)		
N3	2 (0.8%)	1 (0.5%)	1 (1.7%)		
not known	5 (2.1%)	3 (1.6%)	2 (3.4%)		
**Metastases at diagnosis**				**<0.001**	
M0	212 (89%)	169 (93.8%)	43 (74.1%)		
M1	23 (9.7%)	9 (5%)	14 (24.1%)		
not known	3 (1.2%)	2 (1.1%)	1 (1.7%)		
**Tumor stage at diagnosis**				**<0.001**	
Stage Ia	118 (49.6%)	104 (57.7%)	14 (24.1%)		
Stage Ib	23 (9.7%)	18 (10%)	5 (8.6%)		
Stage IIa	22 (9.7%)	13 (7.2%)	9 (15.5%)		
Stage IIb	7 (2.9%)	5 (2.7%)	2 (3.4%)		
Stage IIIa	16 (6.7%)	10 (5.5%)	6 (10.3%)		
Stage IIIb	2 (0.8%)	1 (0.5%)	1 (1.7%)		
Stage IV	24 (10.1%)	9 (5%)	15 (25.8%)		
not known	26 (10.9%)	20 (11.1%)	6 (10.3%)		

Values are medians (IQR) for continuous variables or N (%) for categorical variables. Percentages are shown for the whole cohort, unless otherwise indicated. *p*-values indicate the difference between TC and AC. (a) 92 patients (38.6%) with missing information, *n* = 146. (b) 82 patients (34.4%) with missing information, *n* = 156.

**Table 2:** Diagnostic evaluation.

	All (*n* = 238)	Typical Carcinoid (*n* = 180)	Atypical Carcinoid (*n* = 58)	*p* Value
**Imaging**				
CT scan	204 (85.7%)	155 (86.1%)	49 (84.4%)	0.58
X-ray	111 (46.6%)	90 (50%)	21 (36.2%)	0.10
MRI	31 (13%)	17 (9.4%)	14 (24.1%)	**0.014**
FDG PET/CT	91 (38.2%)	64 (35.5%)	27 (46.5%)	0.30
Somatostatin Receptor Scintigraphy	19 (8%)	12 (6.6%)	7 (12%)	0.36
^68^Ga-DOTATATE/TOC PET/CT	54 (22.6%)	34 (18.8%)	20 (34.4%)	**0.045**
Endoscopy	55 (23.1%)	41 (22.7%)	14 (24.1%)	0.80
**Histology**				
Chromogranin A (positive)	185 (77.7%)	141 (78.3%)	44 (75.8%)	0.54
Synaptophysin (positive)	197 (82.8%)	148 (82.2%)	49 (84.4%)	0.96
SST_2A_ (positive)	45 (18.9%)	34 (18.8%)	11 (18.9%)	0.31
Transcription factors (positive)	52 (21.8%)	33 (18.3%)	19 (32.7%)	0.059
Cytokeratin (positive)	45 (18.9%)	31 (17.2%)	14 (24.1%)	0.39

Table shows the available diagnostic features that were applied in our study cohort. Percentages are indicated for the whole cohort. *p*-values indicate the difference between TC and AC. Additional imaging techniques were used, such as bone scintigraphy (*n* = 6), endoscopic sonography (*n* = 5), sonography (*n* = 13), L-Dopa PET/CT (*n* = 1), perfusion scintigraphy (*n* = 1), and ^177^Lu DOTATOC PET/CT (*n* = 1).

**Table 3:** Therapy and outcome.

	All (*n* = 238)	Typical Carcinoid (*n* = 180)	Atypical Carcinoid (*n* = 58)	*p* Value	
**Surgery as first therapeutical approach**	205 (86.1%)	161 (89.4%)	44 (75.8%)	**<0.001**	(a)
R0 Resection	195 (81.9%)	154 (85.5%)	41 (70.6%)	**0.009**	(b)
Lymphadenectomy performed	148 (62.2%)	112 (62.2%)	36 (62.0%)	0.98	(c)
**Type of Resection**				0.28	
Lobectomy	96 (40.3%)	72 (40%)	24 (41.3%)		
Wedge resection	40 (16.8%)	33 (18.3%)	7 (12%)		
Segmentectomy	33 (13.8%)	27 (15%)	6 (10.3%)		
Bilobectomy	14 (5.9%)	8 (4.4%)	6 (10.3%)		
Bronchial resection	3 (1.2%)	3 (1.6%)	0 (0%)		
Lingula resection	2 (0.8%)	2 (1.1%)	0 (0%)		
Pneumectomy	2 (0.8%)	1 (0.5%)	1 (1.7%)		
Sleeve resection	1 (0.4%)	1 (0.5%)	0 (0%)		
Lung surgery	8 (3.3%)	8 (4.4%)	0 (0%)		
not known	3 (1.2%)	3 (1.6%)	0 (0%)		
Lobar resection	112 (47%)	81 (45%)	31 (53.4%)	**0.012**	(d)
**Additional Therapies**					
PRRT	17 (7.1%)	4 (2.2%)	13 (22.4%)	**<0.001**	
Somatostatin Analogues	17 (7.1%)	8 (4.4%)	9 (15.5%)	**0.012**	(e)
External irradiation	12 (5%)	2 (1.1%)	10 (17.2%)	**<0.001**	
SIRT	3 (1.2%)	1 (0.5%)	2 (3.4%)	0.086	
Chemotherapy	17 (7.1%)	4 (2.2%)	13 (22.4%)	**<0.001**	(f)
Targeted therapy	12 (5%)	4 (2.2%)	8 (13.7%)	**0.002**	(g)
Ablative therapy	8 (3.3%)	3 (1.6%)	5 (8.6%)	**0.042**	(h)
**Follow-up (months)**	49.7 (16.5–84.8)	50.1 (15.4–87.9)	48 (20.1–76.8)	0.73	
**Outcome at last follow-up**				**<0.001**	
Aftercare finished	38 (15.9%)	35 (19.4%)	3 (5.1%)		
Complete remission	120 (50.4%)	100 (55.5%)	20 (34.4%)		
Partial remission	1 (0.4%)	0 (0%)	1 (1.7%)		
Stable disease	13 (5.4%)	6 (3.3%)	7 (12%)		
Progressive disease	10 (4.2%)	4 (2.2%)	6 (10.3%)		
Death	33 (13.8%)	17 (9.4%)	16 (27.5%)		
Lost to follow-up	8 (3.3%)	6 (3.3%)	2 (3.4%)		
not known	15 (6.3%)	12 (6.6%)	3 (5.1%)		

Values are median (IQR) for continuous variables or N (%) for categorical variables. Percentages are indicated for the whole cohort. PRRT = peptide receptor radio therapy. SIRT = selective internal radiotherapy. (a) First therapeutical approach not known in 16 patients (6.7%). (b) Result of resection not known in 5 patients (2.1%). (c) Status of lymph node resection not known in 52 patients (21.8%). (d) Extent of resection not known in 11 patients (4.6%) (lobar versus sublobar); no surgery performed in 36 patients (15.1%). (e) The somatostatin analogues used were either Octreotide (*n* = 7) or Lanreotide (*n* = 10). (f) The medications used for chemotherapy were Cabezitabin and Temozolomid (*n* = 5), Temozolomid mono (*n* = 3), Cisplatin and Pemetrexed (*n* = 1), Pemetrexed mono (*n* = 1), Oxaliplatin (*n* = 1), 5-Fluorouracil and Bevacicumab (*n* = 2), Cisplatin and Etoposid (*n* = 3), Carboplatin (*n* = 1), and Carboplatin and Etoposid (*n* = 1). (g) Twelve patients had Everolimus as targeted therapy; one patient had Sunitinib in combination with Everolimus. (h) Ablative therapy consists of cryotherapy (*n* = 1), embolization (*n* = 2), laser-induced thermo-therapy (LITT) (*n* = 1), and radio frequency ablation (RFA) (*n* = 4).

**Table 4:** Comparison before and after 2016.

	Before 2016 (*n* = 155)	After 2016 (*n* = 83)	*p* Value	
Typical Carcinoids	117 (75.4%)	63 (75.9%)	0.94	
^68^Ga-DOTATATE/TOC PET	25 (16.1%)	29 (34.9%)	**<0.001**	(a)
Somatostatin Receptor Szintigraphy	20 (12.9%)	1 (1.2%)	**<0.001**	(a)
FDG PET/CT	72 (46.4%)	19 (22.8%)	**<0.001**	(a)
assessment of Ki67	89 (57.4%)	67 (80.7%)	**<0.001**	
assessment of SSTR	49 (31.6%)	39 (46.9%)	**0.019**	
Lymphadenectomy	83 (53.5%)	65 (78.3%)	**<0.001**	(b)

All values are N (%). Percentages are indicated for the whole cohort. SSTR = somatostatin receptor. (a) Used imaging techniques not known in 12 patients (5%): 3 (1.9%) before 2016 and 9 (10.8%) after 2016. (b) Status of lymph node resection not known in 52 patients (21.8%): 51 (32.9%) before 2016 and 1 (1.2%) after 2016.

## Data Availability

De-identified individual participant data that underlie the results reported in this article will be shared upon publication to researchers who provide a methodologically sound proposal to achieve the aims in the approved proposal. Proposals should be directed to the corresponding author. To gain access, data requestors will need to sign a data access agreement.
